# Supplementary material for: Preeclampsia in mice carrying fetuses with APOL1 risk variants
Source: bioRxiv. 2024 Mar 25:2024.03.20.586039. Preprint. [Version 2] doi: 10.1101/2024.03.20.586039 (PMC11722342; doi:10.1101/2024.03.20.586039)
Supplement: Supplement 1 [file media-1.pdf]

Figure S1

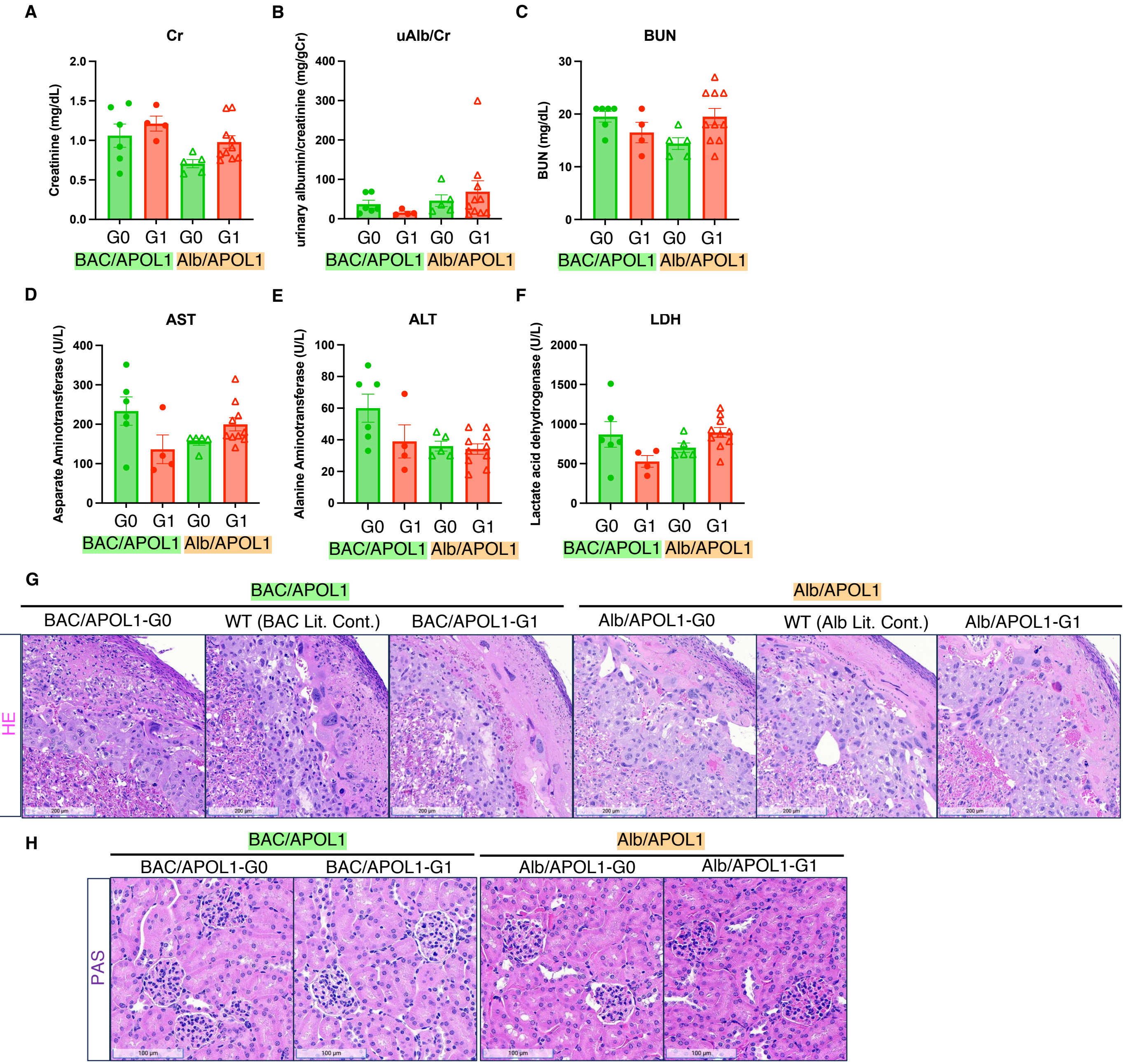

Supplemental Figure 1. Additional phenotypes and pathologies of BAC/APOL1 and Alb/APOL1 models
(**A-F**) Serum creatinine, urinary albumin-to-creatinine ratio, blood urea nitrogen, AST, ALT, and LDH of dams identified by fetal genotype
(**G**) Representative images of HE staining of placentas (**H**) Representative images of PAS staining of maternal kidneys

Figure S2

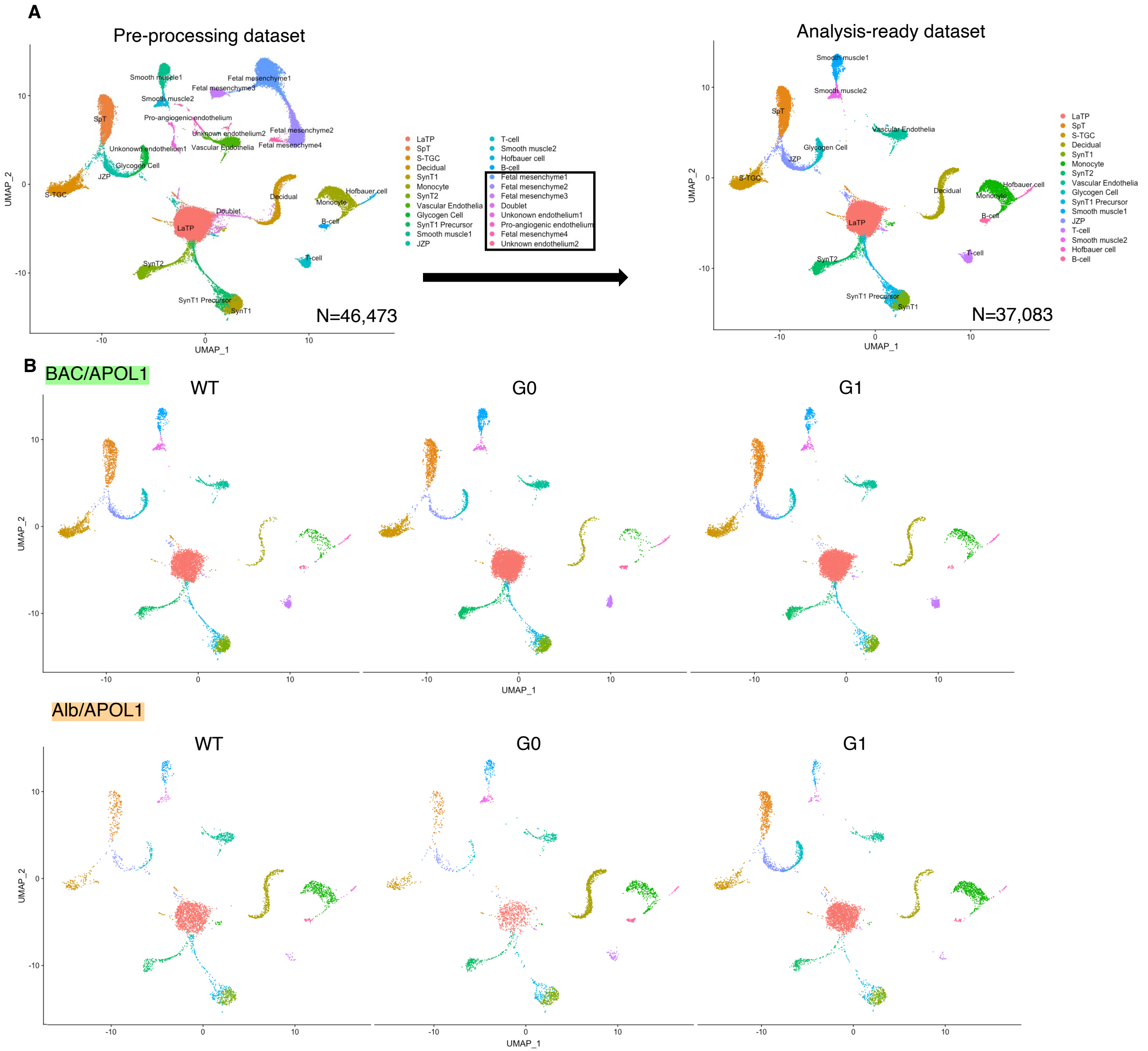

Supplemental Figure 2. Supplemental single-nuclear RNA-seq data, pre-processing and UMAP
(**A**) UMAP plot of pre-processing dataset (N=46,473) and analysis-ready dataset (N=37,083) (**B**) UMAP plots separated by each model

Figure S3

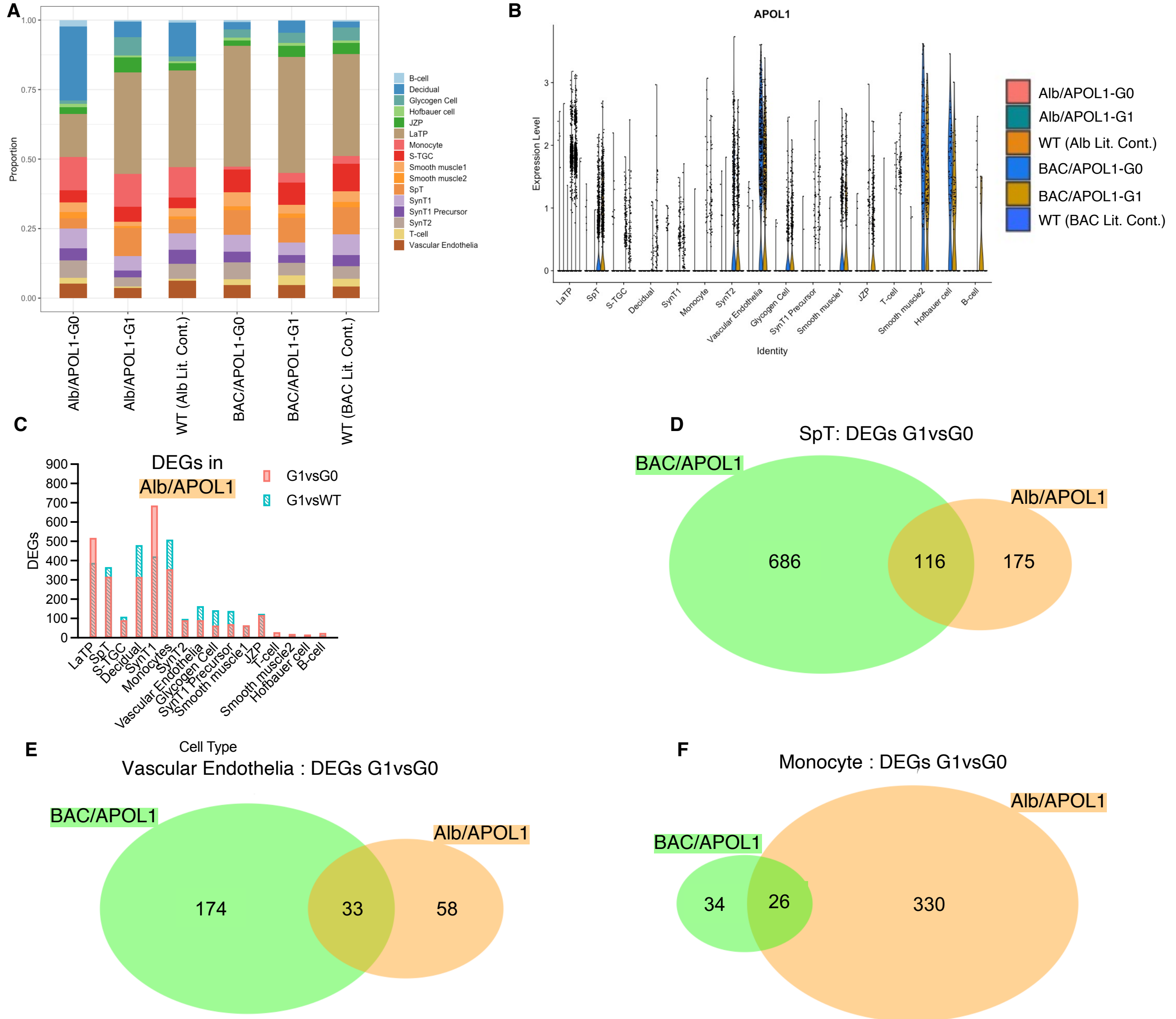

Supplemental Figure 3. Additional single-nuclear RNA-seq data

(**A**) Ratio of nuclei grouped to each cluster by each sample (**B**) Violin plot showing APOL1 expression in each sample in each cluster

(**C**) Number of DEGs in each cluster, comparing APOL1 genotype (G1 vs G0 and G1 vs WT) in Alb/APOL1 model.

(**D-F**) Venn diagram of DEGs of SpT, vascular endothelia, and monocyte, comparing APOL1 genotype (G1 vs G0) in both BAC/APOL1 and Alb/APOL1 model

Figure S4

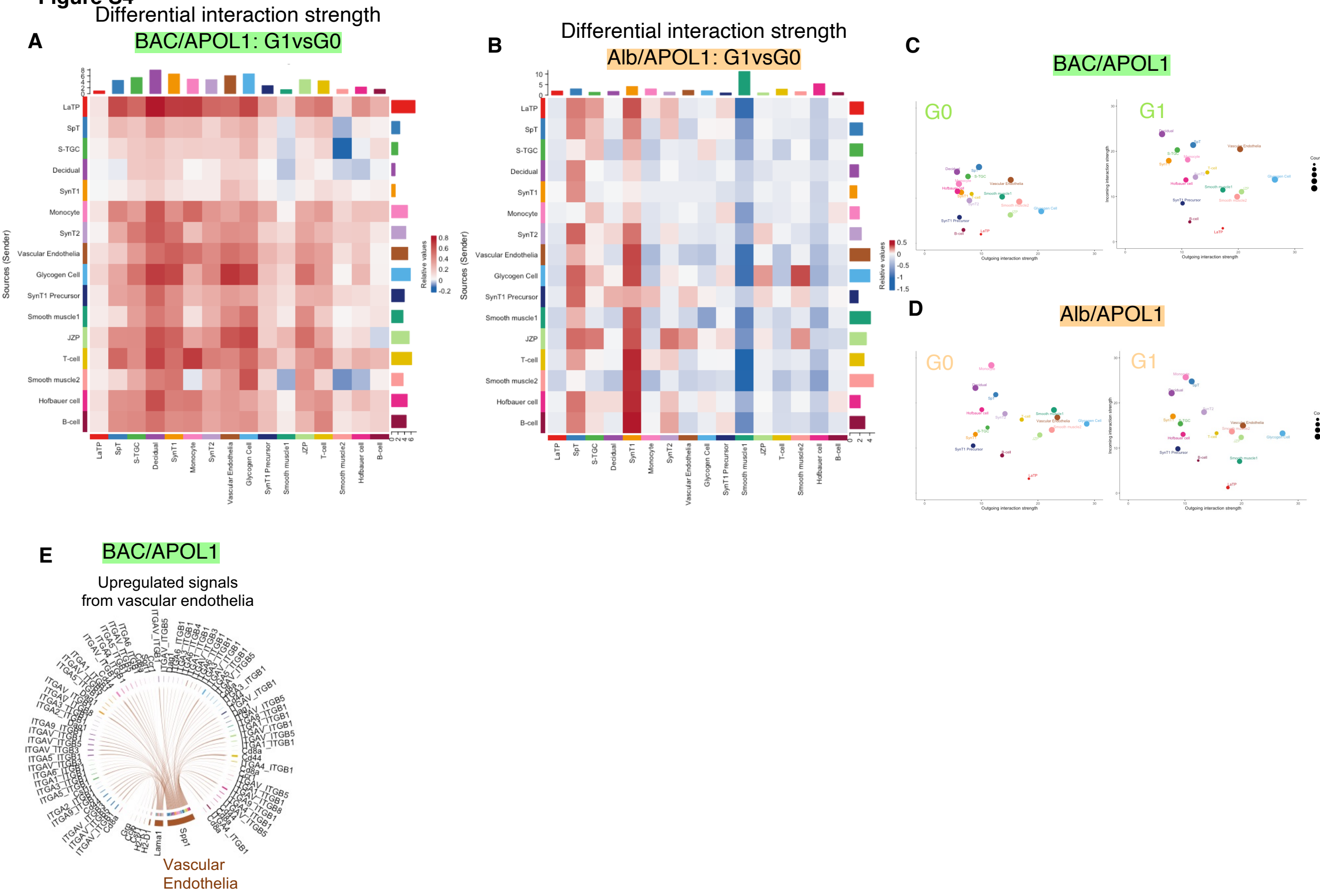

Supplemental Figure 4. Additional cell-cell interaction analysis data

(**A,B**) Heatmaps showing differential interaction strength among cell types comparing APOL1-G1 vs G0 in each of BAC/APOL1, Alb/APOL1 models

(**C,D**) 2D plot showing incoming and outgoing interaction strength in each cell type comparing APOL1-G1 vs G0 in each of BAC/APOL1, Alb/APOL1 models

(**E**) Chord plot showing upregulated signals from vascular endothelia in BAC/APOL1 model

Figure S5

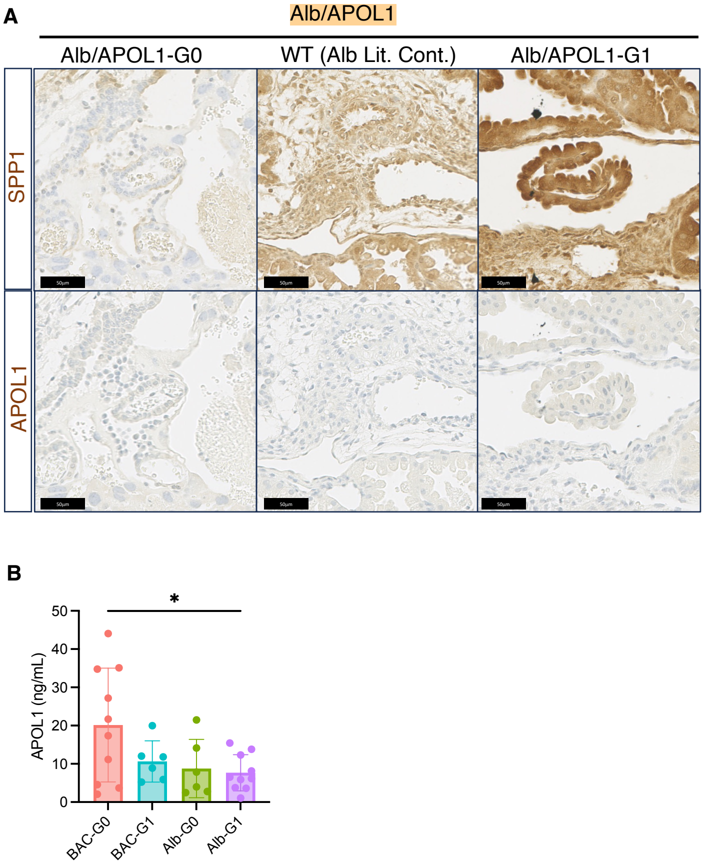

Supplemental Figure 5. Additional IHC of placenta from Alb/APOL1 model and plasma APOL1 level of dams.
(**A**) Representative IHC images of SPP1 and APOL1 of Alb/APOL1 model placenta (**B**) Plasma APOL1 level measured in dams
